# STAT Family Protein Expression and Phosphorylation State during moDC Development Is Altered by Platinum-Based Chemotherapeutics

**DOI:** 10.1155/2019/7458238

**Published:** 2019-06-11

**Authors:** Nienke de Haas, Coco de Koning, Stefania di Blasio, Georgina Flórez-Grau, I. Jolanda M. de Vries, Stanleyson V. Hato

**Affiliations:** Department of Tumor Immunology, Radboud Institute for Molecular Life Sciences, Radboud University Medical Center, Nijmegen, Netherlands

## Abstract

The STAT signaling pathway is important in dendritic cell (DC) development and function. Tumor cells can induce STAT signaling, thereby inhibiting DC maturation and immunostimulatory functions, leading to hampered efficacy of DC-based immunotherapies. Platinum-based chemotherapeutics can inhibit STAT signaling, thereby making them an interesting tool to improve DC development and function. In this study, we provide a comprehensive overview of STAT expression and phosphorylation during DC differentiation and maturation and investigate the effects of platinum drugs on STAT signaling during these processes. Monocytes were differentiated into monocyte-derived DCs (moDCs) with IL-4 and GM-CSF and matured with cytokines or TLR ligands. STAT expression and phosphorylation were analyzed by western blotting, and moDC viability and phenotype were analyzed by flow cytometry. Platinum drugs were added at day 3 of differentiation or at the start of maturation to investigate regulation of the STAT signaling pathway. All STAT proteins were expressed during moDC differentiation and STAT1, STAT5, and STAT6 were phosphorylated. No significant changes occurred in the expression and phosphorylation state of the STAT proteins during differentiation. After maturation with TLR ligands, the expression of STAT1 increased, but other STAT proteins were not affected. Phosphorylation of STAT1 and STAT3 increased during maturation, where TLR ligands induced significantly higher levels of phosphorylation than cytokines. Platinum drugs cisplatin and oxaliplatin significantly inhibited phosphorylation of STAT6 during differentiation and maturation. Treatment did not affect the phenotype or viability of the cells. As STAT6 is an important regulator of DC function, these findings suggest a role for platinum-based chemotherapeutics to enhance DC function via inhibition of STAT signaling, thereby potentially enhancing efficacy of DC-based immunotherapies.

## 1. Introduction

The signal transducer and activator of transcription (STAT) signaling pathway is regulated by a family of 7 STAT proteins that can be induced by over 50 cytokines, growth factors, and hormones. Binding of these factors to their receptors initiates the phosphorylation of receptor-associated Janus kinases (JAKs). Phosphorylated JAKs subsequently form a docking site for STAT proteins where they are phosphorylated. Phosphorylated STAT proteins can then directly bind to DNA and regulate gene transcription [1]. Additionally, nonreceptor tyrosine kinases, like the Src kinase family, can induce STAT signaling directly in the cytoplasm [2]. Mutations in proteins of the STAT signaling pathway have been linked to many human disorders and often result in some form of immunodeficiency, indicating their important role in modulating immune cell function [3, 4]. For example, STAT3 was shown to be important in the development of T cell memory [5], but it also induces differentiation and functional regulation of immune inhibitory myeloid-derived suppressor cells (MDSCs) [6]. Furthermore, activation of STAT proteins is critical for differentiation, phenotype, and function of dendritic cells (DCs), regulating both immunostimulatory and inhibitory mechanisms [7–9].

Dendritic cells are the antigen-presenting cells of the immune system that are present in all tissues, where they scan their environment for signs of danger in the form of protein antigens. After encounter with an antigen, they mature, migrate to lymph nodes, and present antigens to lymphoid cells, thereby efficiently inducing naïve T cells into antigen-specific effector T cells. This property makes them an attractive tool for stimulating antitumor immunity using immunotherapeutic approaches, such as DC vaccination [10]. The two main blood-circulating DC subsets in humans are conventional DCs and plasmacytoid DCs (pDCs). These two subsets exert different functions in immune responses, as conventional DCs have high cross-presentation capacity and pDCs are important in antiviral immunity by producing interferon-*α* [11]. Conventional DCs mainly develop from myeloid precursor cells originating from the bone marrow, which differentiate into immature myeloid DCs in the spleen. pDCs, on the other hand, originate from a lymphoid progenitor cell and differentiate in the bone marrow [12]. Due to the low frequency of blood-circulating DCs in human peripheral blood, monocyte-derived DCs (moDCs) are routinely used as an *in vitro* model to study the development and function of DCs. MoDCs are generated from peripheral blood monocytes, by the addition of granulocyte-macrophage colony-stimulating factor (GM-CSF) and interleukin (IL)-4. They resemble naturally occurring blood DCs in their ability to upregulate costimulatory molecules in response to maturation stimuli and present captured antigens to T cells [13].

Different STAT proteins are involved in the regulation of DC development and functional maturation. STAT1 regulates pDC generation from murine progenitor cells [14]. Moreover, STAT1 is required for the induction of antigen-specific cytotoxic T cell activity by DCs [7, 15]. STAT5 regulates the differentiation of DCs by inducing expansion of conventional DCs and inhibiting development of pDCs [16, 17]. STAT5 is also required for DC activation through upregulation of costimulatory molecules and enhanced chemokine production [18]. Interestingly, STAT3 and STAT6 have both stimulatory and inhibitory effects on DCs. Several studies have shown that STAT3 and STAT6 induce the differentiation of progenitor cells into immature DCs [17, 19–21]. However, STAT3 induction by tumor-derived factors, such as IL-6, inhibits DC maturation [22, 23]. Moreover, STAT3 and STAT6 negatively regulate the immunostimulatory function of DCs by inducing the expression of the inhibitory molecules programmed death-ligand (PD-L) 1 and 2 [8, 9]. Additionally, STAT3 has been described to modulate the development of tolerogenic DCs, inhibit the expression of HLA-DR and costimulatory molecules CD80 and CD86, and reduce the ability of DCs to prime interferon-*γ* production by T cells [24–27]. These observations propose STAT signaling as a possible target to modulate DC development and function.

Several studies have shown that anticancer platinum drugs, including oxaliplatin and cisplatin, are regulators of the STAT signaling pathway. Platinum-based drugs can inhibit phosphorylation of STAT1, 2, 3, 5, and 6 in cancer cells, by specifically blocking the SH2 domain of the STAT proteins, which functions as a docking site of the STAT protein to its receptor, thereby inhibiting *de novo* STAT phosphorylation [28–30]. Additionally, platinum drugs affect STAT6 phosphorylation in moDCs [9]. Treatment of colon cancer patients with DC vaccination in combination with oxaliplatin resulted in functional tumor antigen-specific T cell responses, as well as improved nonspecific T cell proliferation [31]. This effect is possibly caused by the inhibitory effect of oxaliplatin on STAT signaling, as exposure of mature moDCs to platinum drugs *in vitro* downregulated STAT6-dependent PD-L2 expression, thereby enhancing their ability to induce T cell proliferation [9]. Altogether, these observations emphasize the potential role of platinum drugs in modulating STAT signaling to enhance the function and possibly the development of DCs.

Despite the wealth of evidence showing the importance of STAT signaling in DC development and function, a comprehensive expression profile of STATs during differentiation and maturation of (mo)DCs is still lacking. Given the reported inhibitory effect of platinum drugs on STAT6 phosphorylation in moDCs, we aim to investigate the role of oxaliplatin and cisplatin on the expression and phosphorylation of different STAT proteins during moDC development. We provide an overview of the regulation of the STAT signaling pathway during moDC differentiation and maturation using either cytokines or TLR ligands as maturation stimuli. Furthermore, our results show that platinum drugs significantly inhibit phosphorylation of STAT6 during both moDC differentiation and maturation and we observed a trend in inhibition of STAT3 phosphorylation during maturation, which was however not statistically significant. These findings suggest that platinum drugs could be a potential tool to enhance the function of DCs by inhibition of STAT signaling and thereby possibly induce the efficacy of DC-based vaccines.

## 2. Material and Methods

### 2.1. MoDC Generation and Culture

Peripheral blood mononuclear cells (PBMCs) were isolated from healthy donor blood (Sanquin, Nijmegen, Netherlands) by density gradient centrifugation using lymphoprep (Axis-Shield). Adherent cells were obtained as previously described [32]. Immature DCs were generated by culturing the adherent cells in X-VIVO medium (Lonza) containing 2% human serum (Sanquin), with IL-4 (300 U/ml) and GM-CSF (450 U/ml; both CellGenix) for 6 days. On day 6, DCs were maturated with either a cytokine cocktail containing prostaglandin E_2_ (PGE_2_; 10 *μ*g/ml; Pfizer), tumor necrosis factor (TNF)-*α* (10 ng/ml), IL-1*β* (5 ng/ml), and IL-6 (15 ng/ml; all CellGenix) or with toll-like receptor (TLR)3 and TLR7/8 ligands, poly[I:C] (20 *μ*g/ml; Enzo), and R848 (4 *μ*g/ml; InvivoGen), respectively, for 24 hours.

### 2.2. Platinum Treatment

MoDCs were treated with clinically relevant concentrations of platinum drugs during differentiation or maturation [9]. Oxaliplatin (5 mg/ml, TEVA), at a concentration of 4 *μ*g/ml or 7 *μ*g/ml, or cisplatin (1 mg/ml, Accord), at a concentration of 2.5 *μ*g/ml or 5 *μ*g/ml, was added to the culture medium, either at day 3 to day 4 or at day 6 to day 7, to investigate the effect on differentiation and maturation, respectively. Cells were harvested by adding 4°C PBS and were washed with PBS. Around 1 million cells were stored at 4°C for further flow cytometry analysis the same day and all other cells were snapfrozen and stored at -80°C until lysis. Purity of the harvested moDCs was on average 88%.

### 2.3. Flow Cytometry Analysis

Expression levels of maturation markers and costimulatory molecules were evaluated using flow cytometry. Between 50,000 and 80,000 cells per well were first stained for cell viability using Fixable Viability Dye eFluor450 (1 : 2000 dilution; eBioscience) in PBS for 20 minutes. Thereafter, single stainings were performed with the antibodies shown in Table 1 (all Miltenyi) for 30 minutes in autoMACS Running Buffer (Miltenyi). Antibodies were properly titrated to obtain an optimal signal to noise ratio. Cells were analyzed using a FACSVerse flow cytometer (BD). Quality control of the flow cytometer's performance and coefficient of variation (CV) values were monitored on a day-to-day basis using CS&T beads (BD). Data was analyzed using FlowJo V10 software (Treestar) by first gating the moDC population based on forward and sideward scatter and selecting live cells that were negative for Fixable Viability Dye eFluor450, whereafter data was visualized using GraphPad Prism software.

### 2.4. Western Blot Analysis

STAT protein expression and phosphorylation status was assessed by western blotting. Cell pellets were lysed in lysis buffer (pH 7.8) containing 50 mM Tris base, 1 mM EDTA, 150 mM NaCl (all Merck), 1% NP40 (Roche Diagnostics), 1 : 100 phosphatase inhibitor cocktail 2 and 3 (Merck), 1X protease inhibitor cocktail, and 1X PhosSTOP (both Roche Diagnostics). Before polyacrylamide gel electrophoresis, Laemmli sample buffer (Bio-Rad) was added 1 : 5 to cell lysates containing 25 *μ*g of protein for STAT1, 3, 5, and 6, 100 *μ*g for pSTAT1, 3, and 6, or 200 *μ*g for pSTAT5. Samples were fractioned by electrophoresis in 8% SDS-PAGE gels, using 30% 37.5 : 1 Acrylamide/Bis solution (Bio-Rad) and further processed for western blot analysis [33]. After blocking, the primary antibodies in Table 2 were used for overnight staining at 4°C.

After washing, membranes were incubated with 1 : 5000 goat-anti-rabbit IRDye800 (LI-COR Biosciences, order no. 926-32211), 1 : 5000 goat-anti-mouse IRDye680 (LI-COR Biosciences, order no. 926-32220), and 1 : 5000 goat-anti-rat IRDye680 (Invitrogen, order no. A21096) as secondary antibodies and analyzed with the Odyssey Imaging system (LI-COR Biosciences).

### 2.5. Statistical Analysis

**Figure 1:**
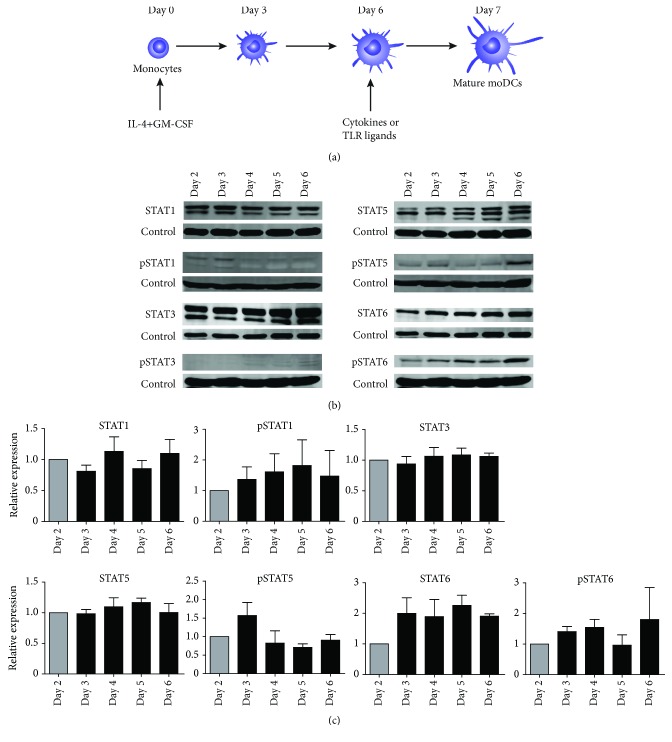
MoDC development protocol and expression and phosphorylation of STAT proteins during monocyte differentiation into moDCs. (a) Experimental design for differentiation and maturation of monocytes into mature moDCs. (b) Expression and phosphorylation of STAT1, STAT3, STAT5, and STAT6 on days 2 to 6, with tubulin used as loading control. One representative band of 3 donors is shown. (c) Quantification of STAT expression and phosphorylation, normalized to tubulin, shown as relative expression to day 2 moDCs (*n* = 3). Data are depicted as mean + SEM.

**Figure 2:**
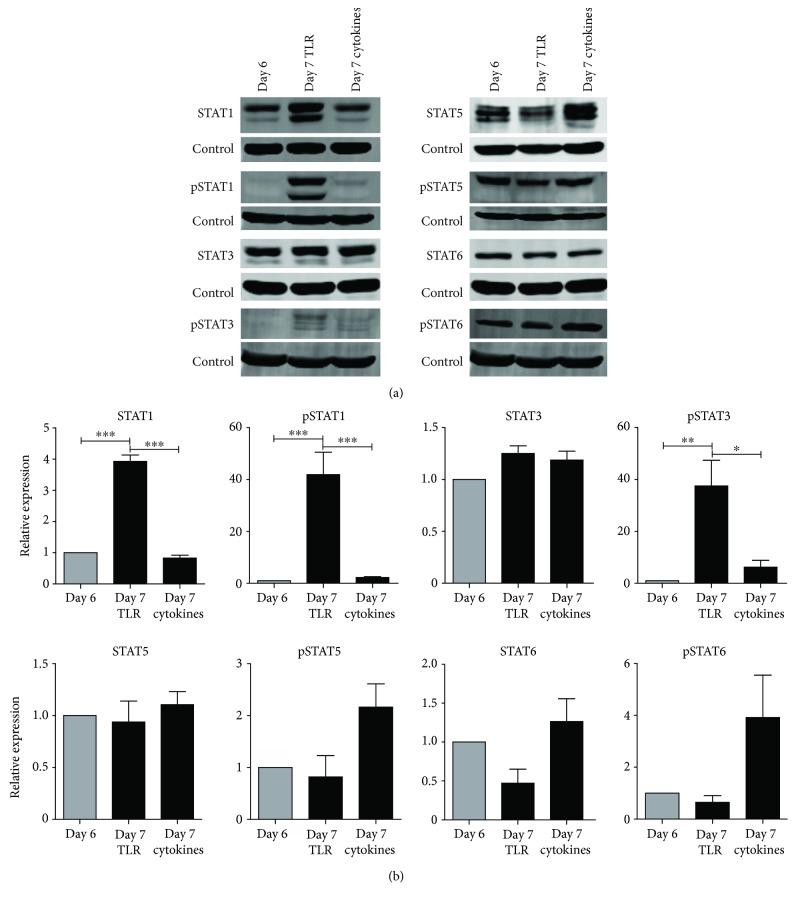
Expression and phosphorylation of STAT proteins during moDC maturation. (a) Expression and phosphorylation of STAT1, STAT3, STAT5, and STAT6 on day 6, before addition of maturation stimuli, and day 7 after maturation with TLR ligands or cytokines, with tubulin used as loading control. One representative band of 4 donors is shown. (b) Quantification of STAT expression and phosphorylation, normalized to tubulin, shown as relative expression to day 6 moDCs (*n* = 4). Data are depicted as mean + SEM. ^∗^ *P* < 0.05; ^∗∗^ *P* < 0.01; ^∗∗∗^ *P* < 0.001.

**Figure 3:**
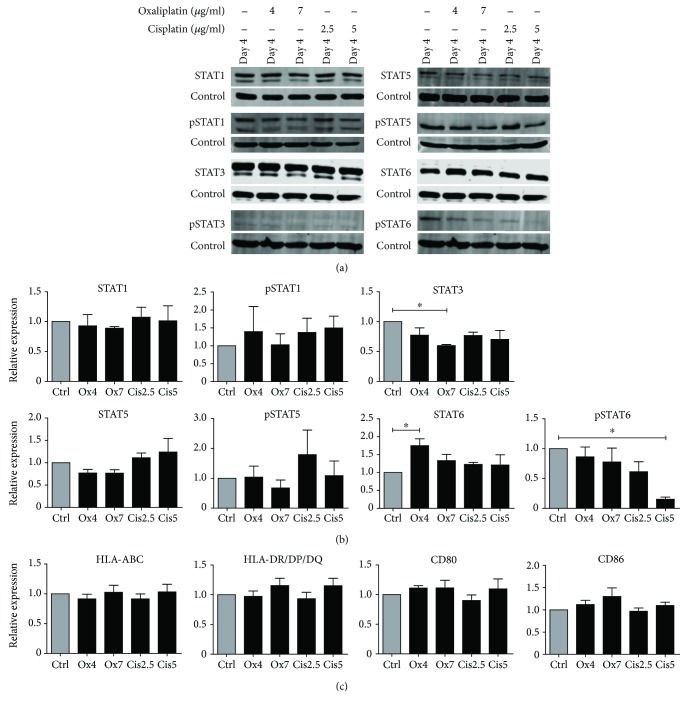
Expression and phosphorylation of STAT proteins and phenotype of moDCs during differentiation in the presence or absence of platinum drugs. (a) Expression and phosphorylation of STAT1, STAT3, STAT5, and STAT6 on day 4 of differentiation in the presence or absence of oxaliplatin (4 *μ*g/ml or 7 *μ*g/ml) or cisplatin (2.5 *μ*g/ml or 5 *μ*g/ml), with tubulin used as loading control. One representative band of 3 donors is shown. (b) Quantification of STAT expression and phosphorylation, normalized to tubulin, shown as relative expression to untreated control cells (*n* = 3). Data are depicted as mean + SEM. (c) Expression of HLA-ABC and HLA-DR/DQ/DQ and costimulatory molecules CD80 and CD86 by moDCs treated with oxaliplatin (4 *μ*g/ml or 7 *μ*g/ml) or cisplatin (2.5 *μ*g/ml or 5 *μ*g/ml) during differentiation as compared to untreated moDCs. The mean fluorescence intensities (MFIs) of live cells are shown as mean fold of untreated control cells + SEM (*n* = 4). ^∗^ *P* < 0.05.

**Figure 4:**
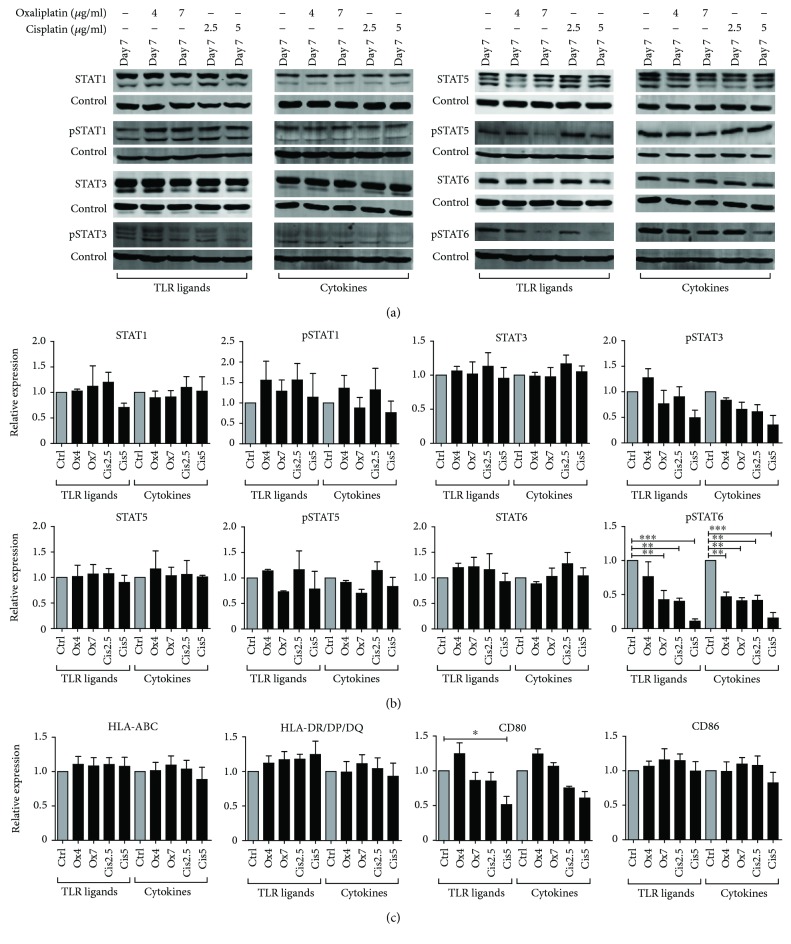
Expression and phosphorylation of STAT proteins and phenotype of moDCs during maturation in the presence or absence of platinum drugs. (a) Expression and phosphorylation of STAT1, STAT3, STAT5, and STAT6 on day 7 after maturation in the presence or absence of oxaliplatin (4 *μ*g/ml or 7 *μ*g/ml) or cisplatin (2.5 *μ*g/ml or 5 *μ*g/ml), with tubulin used as loading control. One representative band of 3 donors is shown. (b) Quantification of STAT expression and phosphorylation, normalized to tubulin, shown as relative expression to untreated control cells (*n* = 3). Data are depicted as mean + SEM. (c) Expression of HLA-ABC and HLA-DR/DQ/DQ and costimulatory molecules CD80 and CD86 by moDCs treated with oxaliplatin (4 *μ*g/ml or 7 *μ*g/ml) or cisplatin (2.5 *μ*g/ml or 5 *μ*g/ml) during maturation as compared to untreated moDCs. The MFIs of live cells are shown as mean fold of untreated control cells + SEM (*n* = 4 (TLR maturation) or *n* = 3 (cytokine maturation)). MoDCs matured with TLR ligands and moDCs matured with cytokines are obtained from different donors. ^∗^ *P* < 0.05; ^∗∗^ *P* < 0.01; ^∗∗∗^ *P* < 0.001.

Western blot quantification and phenotype data are normalized to the loading control and are depicted as relative expression to day 2 or day 6 + SEM (Figures 1 and 2) or as relative expression to control + SEM (Figures 3 and 4). Statistical testing was performed using one-way ANOVA followed by Bonferroni's multiple comparison test comparing STAT expression and phosphorylation at all analyzed days to each other in Figures 1 and 2 and the platinum conditions to their control in Figures 3 and 4.

## 3. Results

### 3.1. STAT Expression and Phosphorylation during moDC Differentiation

The expression and tyrosine phosphorylation state of STAT proteins during differentiation of monocytes into moDCs was assessed by western blotting. Monocytes were cultured in medium containing IL-4 and GM-CSF for 6 days. Medium was refreshed on day 3 (Figure 1(a)). The expression and phosphorylation of STAT1, STAT3, STAT5, and STAT6 were analyzed on moDCs at days 2 to 6 of the differentiation process. At day 2, expression of all total STAT proteins was observed and remained at a similar level until day 6 (Figures 1(b) and 1(c)). Additionally, the upper band of STAT5, which is the *α*-isoform, increased during differentiation. Stable levels of phosphorylated STAT1 were observed from day 2 to day 6 and phosphorylation of STAT3 was weak or not detectable. Phosphorylation of STAT5 slightly decreased on days 4, 5, and 6 when compared to day 3 and STAT6 phosphorylation moderately increased at day 6, but these changes were not statistically significant. Furthermore, phosphorylation of STAT2 and STAT4 was not observed during differentiation as well as maturation and is therefore not shown (Supplementary figure 1).

### 3.2. STAT Expression and Phosphorylation during moDC Maturation

On day 6, moDCs were stimulated with either a cytokine cocktail or TLR ligands and mature moDCs were harvested at day 7 (Figure 1(a)). Several maturation markers, such as HLA-ABC and HLA-DR/DP/DQ, and costimulatory markers, CD80 and CD86, were shown to be upregulated on moDCs compared to day 2, indicating the moDCs had a matured phenotype (Supplementary figure 2A, B). The gating strategy for viable moDCs is shown in Supplementary figure 2C. Viability of the cells did not change upon culturing from day 2 until day 7 (Supplementary figure 2D). STAT expression and phosphorylation in moDCs harvested on day 6, before addition of the maturation stimuli, were compared to day 7 after TLR-induced or cytokine-induced maturation (Figures 2(a) and 2(b)). Expression of STAT3, STAT5, and STAT6 did not change after maturation, compared to day 6, whereas STAT1 expression significantly increased after TLR-induced maturation and remained unchanged after cytokine-induced maturation. Furthermore, phosphorylation of STAT1 and STAT3 significantly increased after TLR-induced maturation while only a moderate and not significant increase was observed after cytokine-induced maturation. Both maturation stimuli did not significantly alter STAT5 and STAT6 phosphorylation, although cytokine maturation seemed to induce phosphorylation of STAT5 and STAT6 more than maturation with TLR ligands.

### 3.3. Platinum Drugs Alter Expression and Phosphorylation of STAT6 during moDC Differentiation

To determine the effect of platinum drugs on STAT expression and phosphorylation during monocyte differentiation into moDCs, cells were treated with clinically relevant doses of oxaliplatin (4 *μ*g/ml or 7 *μ*g/ml) or cisplatin (2.5 *μ*g/ml or 5 *μ*g/ml) at day 3 of differentiation.  Figure 3(a) shows the expression and phosphorylation levels of STAT1, STAT3, STAT5, and STAT6 on day 4 of differentiation after 24 hours of platinum treatment. Quantification revealed that expression levels of STAT1 and STAT5 were not significantly altered by the addition of platinum drugs (Figure 3(b)). STAT3 expression was significantly decreased but only by the highest concentration of oxaliplatin, and STAT6 expression was increased but only by the lowest concentration of oxaliplatin. Phosphorylation of STAT1 and STAT5 was also not altered by treatment with oxaliplatin or cisplatin, whereas phosphorylated STAT3 was not detectable at day 4. Phosphorylation of STAT6 was significantly downregulated by cisplatin at a concentration of 5 *μ*g/ml compared to control, and a similar trend was observed for the lowest concentration of cisplatin. The increase observed in the expression of total STAT6 emphasizes the potency of especially cisplatin to specifically inhibit phosphorylation of STAT6. In addition, to investigate the effect of platinum drug treatment on the phenotype of the immature moDCs, expression of HLA-ABC and HLA-DR/DP/DQ and costimulatory molecules CD80 and CD86 was analyzed (Figure 3(c)). These markers were not affected by the platinum drugs, indicating that the treatment does not alter the phenotype of the moDCs. Viability of the moDCs was also not affected by the platinum drugs (Supplementary figure 3A).

### 3.4. Platinum Drugs Alter Phosphorylation of STAT3 and STAT6 during moDC Maturation

To determine the effect of platinum drugs on STAT protein expression and phosphorylation during moDC maturation, oxaliplatin (4 *μ*g/ml or 7 *μ*g/ml) or cisplatin (2.5 *μ*g/ml or 5 *μ*g/ml) was added at day 6, together with the maturation stimuli.  Figure 4(a) shows STAT protein expression levels and phosphorylation state on day 7 of maturation with TLR ligands or cytokines in the presence or absence of platinum drugs. Oxaliplatin and cisplatin did not significantly affect the expression of STAT1, STAT3, STAT5, and STAT6 on day 7 after maturation with either TLR ligands or cytokines (Figures 4(a) and 4(b)). Changes in phosphorylation of STAT1 and STAT5 were observed, although not significant due to high donor variance and no trend could be observed towards upregulation or downregulation of phosphorylation by the platinum drugs. Although it was not a significant trend, a slight decrease in the phosphorylation of STAT3 was observed by the highest concentration of cisplatin in TLR-matured moDCs, as well as by both platinum drugs in cytokine-matured moDCs. Furthermore, treatment of moDCs with oxaliplatin (7 *μ*g/ml) or cisplatin significantly inhibited the phosphorylation of STAT6 at day 7 compared to untreated matured moDCs. The lowest concentration of oxaliplatin significantly decreased phosphorylation of STAT6 in cytokine-matured moDCs, but not in TLR-matured moDCs. The expression levels of HLA-ABC and HLA-DR/DP/DQ and costimulatory marker CD86 were not affected by treatment of the mature moDCs (Figure 4(c)). Only the highest concentration of cisplatin significantly inhibited the expression of CD80 on TLR-matured moDCs. Viability of the moDCs was not affected by the treatment (Supplementary figure 3B).

## 4. Discussion

In this study, we provided a comprehensive profile of the expression levels and phosphorylation state of STAT proteins during differentiation of human monocytes into moDCs and during moDC maturation. In addition, we reported that the platinum drugs oxaliplatin and cisplatin inhibit STAT phosphorylation during moDC development. These results provide new insights into STAT protein expression and phosphorylation during moDC development *in vitro* and have implications for the use of platinum-based chemotherapeutics to modulate STAT expression in moDCs, thereby possibly improving their immune stimulatory function.

We observed stable expression of STAT1, STAT3, STAT5, and STAT6 during differentiation. Interestingly, the expression of the *α*-isoform of STAT5 increased during this process, alluding to possible functional differences between STAT5 isoforms in DC differentiation. Expression of phosphorylated STAT1 did not change during differentiation, and phosphorylation of STAT3 was not detected. STAT5 phosphorylation slightly decreased on days 4 to 6 compared to day 3 of the differentiation process, which could possibly be related to the change in STAT5 isoform expression we observed. However, this decrease was not significant. STAT6 phosphorylation was detectable from the start of differentiation and did not also relevantly change upon differentiation. Expression and phosphorylation of STAT proteins can be explained by the presence of IL-4 and GM-CSF in the culture medium during differentiation. In mouse DCs, GM-CSF has been shown to induce expression of STAT1, STAT3, STAT5, and STAT6 [34]. Additionally, the GM-CSF receptor is readily expressed in monocytes, explaining why this effect of GM-CSF is already observed from day 2 onwards [35]. GM-CSF also induced STAT5 phosphorylation in human monocytes, which could explain the observed expression of phosphorylated STAT5 during moDC differentiation [35]. In CD34^+^ human progenitor cells that were differentiated using GM-CSF, high expression of STAT5 and low levels of phosphorylated STAT5 were detected at day 3. STAT5 expression decreased until day 7, whereas phosphorylated STAT5 expression increased over the same period [36]. This is different from our observations that expression and phosphorylation of STAT5 are not significantly altered during differentiation. This interesting difference might be caused by the different origins of the DC subsets and could also be dependent on the difference in cytokine cocktail such as the addition of IL-4 to the cocktail we used. IL-4 directly induces STAT6 phosphorylation in human and mice, and IL-4 and GM-CSF have a synergistic stimulatory effect on STAT1 phosphorylation in mouse studies, which correlates with our observations on STAT1 and STAT6 phosphorylation [37]. Phosphorylation of STAT3 was weak or not detectable during differentiation of monocytes, although it has been described that IL-4 can induce phosphorylation of STAT3 in monocytes [38]. This is an interesting observation as it has been reported that induction of STAT3 phosphorylation in progenitor cells leads to inhibited differentiation into mature DCs and induced development of immune inhibitory MDSCs, which could negatively affect their functionality in DC-based vaccines [39].

Phosphorylation of STAT2 and STAT4 was not observed during either differentiation or maturation, although effects of these STAT proteins on DC development and function have been described. For example, a partial loss-of-function mutation within STAT2 reduces the amounts of both pDCs and conventional DCs in mice and impairs their ability to mature in response to TLR stimulation [40]. Moreover, STAT2 is required for DC-mediated cross-presentation [41]. Similarly, STAT4 induces DC maturation and enhances the capacity of DCs to prime cytotoxic T lymphocytes [42, 43]. The difference in the expression pattern of phosphorylated STAT2 and STAT4 compared to our study is probably due to the use of different cell types and different stimulatory cytokines.

Cisplatin significantly reduced STAT6 phosphorylation at day 4 of differentiation when added at day 3. It has however been described that cisplatin treatment inhibits differentiation of moDCs. After treatment of monocytes during differentiation, the percentage of CD14^+^ undifferentiated monocytes increased and the percentage of CD1a expressing mature cells decreased [44]. This indicates that adding platinum compounds during monocyte differentiation could hamper differentiation of precursor cells into moDCs, possibly through inhibition of STAT6 phosphorylation. However, treatment of moDCs with platinum chemotherapeutics did not affect the expression levels of HLA-ABC, HLA-DR/DP/DQ, CD80, and CD86 nor the percentage of cells positive for these markers (data not shown), indicating that the possible hampering of moDC differentiation by platinum drugs is not caused by phenotypical changes. Furthermore, it shows that the effect of platinum drugs on STAT6 phosphorylation is not related to changes in the phenotype. Nonetheless, given the knowledge that platinum treatment inhibits moDC development, inhibition of STAT6 phosphorylation with platinum drugs during differentiation might ultimately not be beneficial for DCs used in DC-based immunotherapies.

Maturation of moDCs with TLR ligands poly[I:C] and R848 or with a cytokine cocktail consisting of PGE_2_, TNF-*α*, IL-1*β*, and IL-6 had no effect on the expression or phosphorylation of STAT5 and STAT6. The cytokine cocktail slightly induced the phosphorylation of STAT1 and STAT3 compared to day 6. This is probably caused by the presence of IL-6 in the cytokine cocktail, which is a known inducer of phosphorylation of STAT1 and STAT3 [23, 45]. However, the increase in STAT1 and STAT3 phosphorylation was not strong, indicating that other cytokines in the cocktail might influence the effect of IL-6 on phosphorylation or phosphorylation itself. STAT3 expression was unaltered after maturation, whereas STAT1 expression was significantly increased by TLR ligands. Although STAT3 is described to inhibit maturation of moDCs, phosphorylation was strongly increased by maturation with TLR ligands [26]. Phosphorylation of STAT1 was also significantly higher after maturation with TLR3 and TLR7/8 ligands, compared to the cytokine cocktail. The differences in effects of the maturation stimuli on STAT1 expression and phosphorylation can be explained by observations that TLR signaling in DCs induced STAT1 expression and phosphorylation, whereas cytokine maturation with PGE_2_ and TNF-*α* did not induce phosphorylation of STAT1. Additionally, PGE_2_ and TNF-*α* also did not induce phosphorylation of STAT3 and STAT6 [46]. Furthermore, moDCs themselves are also capable of secreting cytokines that could exert autocrine and paracrine effects on STAT expression and phosphorylation. For example, the addition of TLR ligand R848 during differentiation of moDCs increased the phosphorylation of p38 and p42, which drives the production of IL-6 and IL-10 that can in turn induce phosphorylation of STAT3 [8]. This process possibly contributes to the higher level of phosphorylated STAT3 after maturation with TLR ligands, compared to cytokines.

Phosphorylation of STAT1 has a stimulatory effect on DCs and is required for the generation of antigen-specific cytotoxic T cells [15]. As our observations indicate that TLR ligands induce higher STAT1 phosphorylation than cytokines, TLR-induced maturation would be favorable over cytokine-induced maturation to stimulate DC function. This is emphasized by the observation that TLR-matured moDCs have higher T cell stimulatory capacity than moDCs matured with cytokines [47]. However, TLR maturation also induced higher levels of phosphorylated STAT3. STAT3 phosphorylation is related to inhibition of DC function in several studies, as it downregulates expression of HLA-DR and costimulatory molecules CD80 and CD86 [25, 27]. Furthermore, STAT3 induces PD-L1 expression on DCs, which is required for the development of tolerogenic DCs, and reduces the ability of DCs to prime interferon-*γ* production by T cells [8, 24, 26]. STAT6 was also phosphorylated after maturation with TLR ligands and induces PD-L2 expression on moDCs, which is an important T cell inhibitory molecule [9]. Therefore, inhibiting the phosphorylation of these STATs during maturation with TLR ligands could enhance the immunostimulatory function of moDCs. We observed a trend towards inhibition of STAT3 phosphorylation by cisplatin when added at the start of maturation with either cytokines or TLR ligands, although this was not significant. Cisplatin and oxaliplatin significantly inhibited STAT6 phosphorylation during TLR-induced and cytokine-induced maturation, confirming earlier observations that cisplatin and oxaliplatin inhibit STAT6 phosphorylation in moDCs [9]. Inhibition of especially STAT6 phosphorylation by platinum compounds could possibly explain the beneficial effect of oxaliplatin in colon cancer patients that were treated with moDC vaccination. Combination treatment enhanced tumor-antigen T cell responses and improved nonspecific T cell proliferation, which could be due to enhanced DC function by oxaliplatin [31].

Our findings in moDCs indicate that combination therapy with platinum compounds possibly also enhances the efficacy of naturally occurring blood-DC vaccines, which are a promising alternative to moDC vaccines [48]. However, there are indications that platinum drugs have dissimilar effects on different DC subsets. Treatment with platinum drugs induced the T cell proliferative capacity of both TLR-matured moDCs and CD14^+^ monocytes activated with the superantigen staphylococcal enterotoxin B [9, 44]. However, cisplatin stimulated the development of IL-10 producing tolerogenic DCs after TLR stimulation of bone marrow-derived mouse DCs, but there are no indications for a similar effect in human DCs [49]. Furthermore, oxaliplatin impaired the function of TLR9-activated pDCs, possibly by upregulation of PD-L1. The differential effect could be the result of different STAT expression profiles, as STAT3 and STAT6 phosphorylation was not observed during pDC maturation, in contrast to what we observed during moDC maturation. pDCs do however respond differently to oxaliplatin treatment when activated with TLR7 ligands, which partially restored their T cell proliferative capacity. CD1c^+^ myeloid DCs also induced lower T cell proliferation when treated with oxaliplatin during TLR-induced activation [50]. However, oxaliplatin enhanced the T cell stimulatory capacity of CD1c^+^ myeloid DCs when treated in the presence of IL-4 and GM-CSF (data not shown). Therefore, future research is needed to assess if platinum drugs stimulate blood-DC subsets similar to moDCs and what adjuvants are needed to create the optimal environment for platinum drugs to potentiate DC function. Ultimately, platinum drugs could be an interesting potential tool to regulate the STAT signaling pathway in order to improve DC function and enhance the efficacy of DC-based immunotherapies.

## Figures and Tables

**Table 1:** Antibodies used for flow cytometry.

Target	Fluorophore	Clonality	Dilution	Order no.
REA isotype	APC	REA293	1 : 80	130-104-615
HLA-ABC	APC	REA230	1 : 80	130-101-466
HLA-DR/DP/DQ	APC	REA332	1 : 160	130-104-870
mIgG1 isotype	APC	IS5-21F5	1 : 80	130-113-758
CD80	APC	2D10	1 : 80	130-099-710
CD86	APC	FM95	1 : 160	130-114-095

**Table 2:** Primary antibodies used for western blotting.

Target	Origin and clonality	Dilution	Company	Order no.
STAT1	Rabbit-polyclonal	1 : 1000	Cell Signaling	9172
pSTAT1	Rabbit-monoclonal	1 : 1000	Cell Signaling	9167
STAT3	Rabbit-monoclonal	1 : 1000	Cell Signaling	4904
pSTAT3	Rabbit-monoclonal	1 : 1000	Cell Signaling	9145
STAT5	Rabbit-monoclonal	1 : 1000	Cell Signaling	94205S
pSTAT5	Rabbit-monoclonal	1 : 250	Cell Signaling	4322T
STAT6	Rabbit-polyclonal	1 : 1000	Santa Cruz	sc-621
pSTAT6	Mouse-monoclonal	1 : 1000	BD Biosciences	611567
Tubulin	Rat-monoclonal	1 : 1000	Novus	NB100-1639

## Data Availability

All data used to support the findings of this study are included within the article and are available from the corresponding author upon request.

## Supplementary Materials

Supplementary Materials Supplementary figure 1: expression and phosphorylation of STAT2 and STAT4 during moDC differentiation and maturation. A: expression and phosphorylation of STAT2 and STAT4 on days 4 to 6 of differentiation, with actin used as loading control. One representative band of at least 2 donors is shown. B: expression and phosphorylation of STAT2 and STAT4 on day 6, before addition of maturation stimuli and day 8 after maturation with TLR ligands or cytokines, with actin used as loading control. One representative band of at least 3 donors is shown. Supplementary figure 2: phenotype and viability of moDCs during differentiation and maturation. A: representative FACS histogram plots of HLA-ABC, HLA-DR/DP/DQ, CD80 and CD86 expression by moDCs during days 2 and 6 of differentiation and on day 7 after maturation with TLR ligands or cytokines. B: MFI of HLA-ABC, HLA-DR/DP/DQ, CD80 and CD86 expression on moDCs during day 2 and 6 of differentiation and on day 7 after maturation with TLR ligands or cytokines. The MFIs of live cells are shown as mean + SEM (n = 4). C: representative dotplot and FACS histogram of the gating strategy of moDC based on FSC and SSC scatter and subsequent viable cells based on negative staining of fixable viability dye 450 (DCM-450). D: viability of moDCs on days 2 and 6 of differentiation and on day 7 after maturation with TLR ligands or cytokines. Data are depicted as mean + SEM (n = 4). Day 2 and day 6-7 moDCs were obtained from different donors. Supplementary figure 3: viability of moDCs during differentiation and maturation in the presence or absence of platinum drugs. A: viability of moDCs on day 4 of differentiation in the presence or absence of oxaliplatin (4 *μ*g/ml or 7 *μ*g/ml) or cisplatin (2.5 *μ*g/ml or 5 *μ*g/ml). B: viability of moDCs on day 7 after maturation with TLR ligands or cytokines in the presence or absence of oxaliplatin (4 *μ*g/ml or 7 *μ*g/ml) or cisplatin (2.5 *μ*g/ml or 5 *μ*g/ml). Data are depicted as mean + SEM (n = 3). MoDCs matured with TLR ligands and moDCs matured with cytokines were obtained from different donors.
