# Supplementary material for: STAT Family Protein Expression and Phosphorylation State during moDC Development Is Altered by Platinum-Based Chemotherapeutics
Source: J Immunol Res. 2019 Jun 11;2019:7458238. doi: 10.1155/2019/7458238 (PMC6594321; doi:10.1155/2019/7458238)
Supplement: Supplementary Materials — Supplementary figure 1: expression and phosphorylation of STAT2 and STAT4 during moDC differentiation and maturation. A: expression and phosphorylation of STAT2 and STAT4 on days 4 to 6 of differentiation, with actin used as loading control. One representative band of at least 2 donors is shown. B: expression and phosphorylation of STAT2 and STAT4 on day 6, before addition of maturation stimuli and day 8 after maturation with TLR ligands or cytokines, with actin used as loading control. One representative band of at least 3 donors is shown. Supplementary figure 2: phenotype and viability of moDCs during differentiation and maturation. A: representative FACS histogram plots of HLA-ABC, HLA-DR/DP/DQ, CD80 and CD86 expression by moDCs during days 2 and 6 of differentiation and on day 7 after maturation with TLR ligands or cytokines. B: MFI of HLA-ABC, HLA-DR/DP/DQ, CD80 and CD86 expression on moDCs during day 2 and 6 of differentiation and on day 7 after maturation with TLR ligands or cytokines. The MFIs of live cells are shown as mean + SEM (n = 4). C: representative dotplot and FACS histogram of the gating strategy of moDC based on FSC and SSC scatter and subsequent viable cells based on negative staining of fixable viability dye 450 (DCM-450). D: viability of moDCs on days 2 and 6 of differentiation and on day 7 after maturation with TLR ligands or cytokines. Data are depicted as mean + SEM (n = 4). Day 2 and day 6-7 moDCs were obtained from different donors. Supplementary figure 3: viability of moDCs during differentiation and maturation in the presence or absence of platinum drugs. A: viability of moDCs on day 4 of differentiation in the presence or absence of oxaliplatin (4 μg/ml or 7 μg/ml) or cisplatin (2.5 μg/ml or 5 μg/ml). B: viability of moDCs on day 7 after maturation with TLR ligands or cytokines in the presence or absence of oxaliplatin (4 μg/ml or 7 μg/ml) or cisplatin (2.5 μg/ml or 5 μg/ml). Data are depicted as mean + SEM (n = 3). MoDCs matured with T [file 7458238.f1.docx]

**
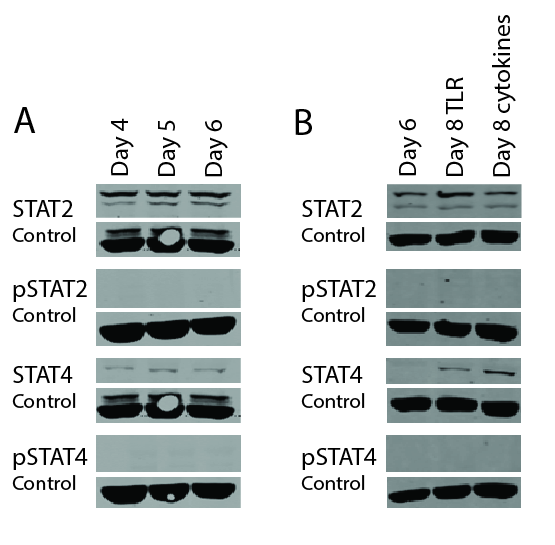
**

**Supplementary figure 1: Expression and phosphorylation of STAT2 and STAT4 during moDC differentiation and maturation. A**: Expression and phosphorylation of STAT2 and STAT4 on days 4 to 6 of differentiation, with actin used as loading control. One representative band of at least 2 donors is shown. **B**: Expression and phosphorylation of STAT2 and STAT4 on day 6, before addition of maturation stimuli and day 8 after maturation with TLR ligands or cytokines, with actin used as loading control. One representative band of at least 3 donors is shown.

**
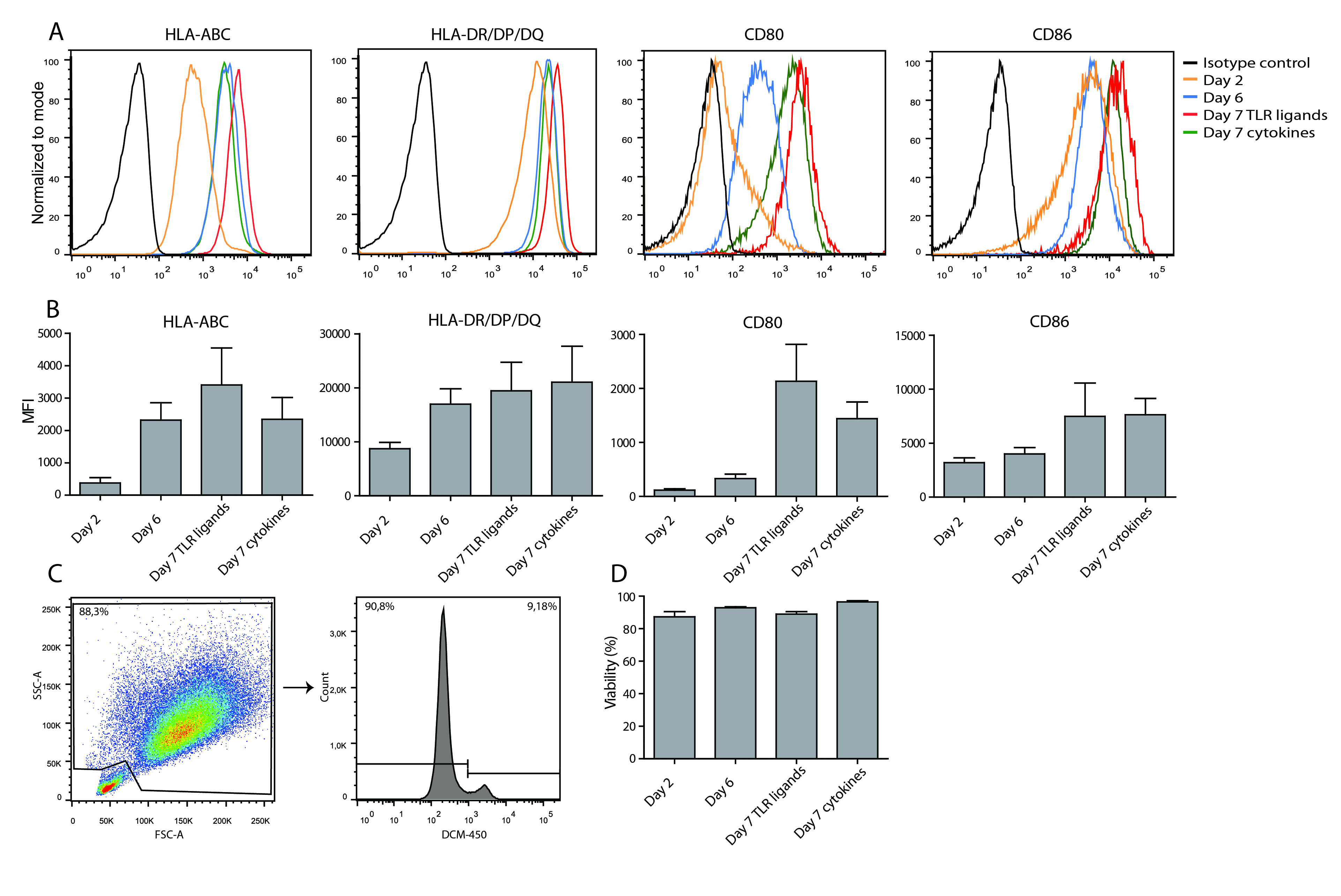
**

**Supplementary figure 2: Phenotype and viability of moDCs during differentiation and maturation. A:** Representative FACS histogram plots of HLA-ABC, HLA-DR/DP/DQ, CD80 and CD86 expression by moDCs during days 2 and 6 of differentiation and on day 7 after maturation with TLR ligands or cytokines. **B:** MFI of HLA-ABC, HLA-DR/DP/DQ, CD80 and CD86 expression on moDCs during days 2 and 6 of differentiation and on day 7 after maturation with TLR ligands or cytokines. The MFIs of live cells are shown as mean + SEM (n=4). **C:** Representative dotplot and FACS histogram of the gating strategy of moDC based on FSC and SSC scatter and subsequent viable cells based on negative staining of fixable viability dye 450 (DCM-450). **D:** Viability of moDCs on days 2 and 6 of differentiation and on day 7 after maturation with TLR ligands or cytokines. Data are depicted as mean + SEM (n=4). Day 2 and day 6-7 moDCs were obtained from different donors.

**
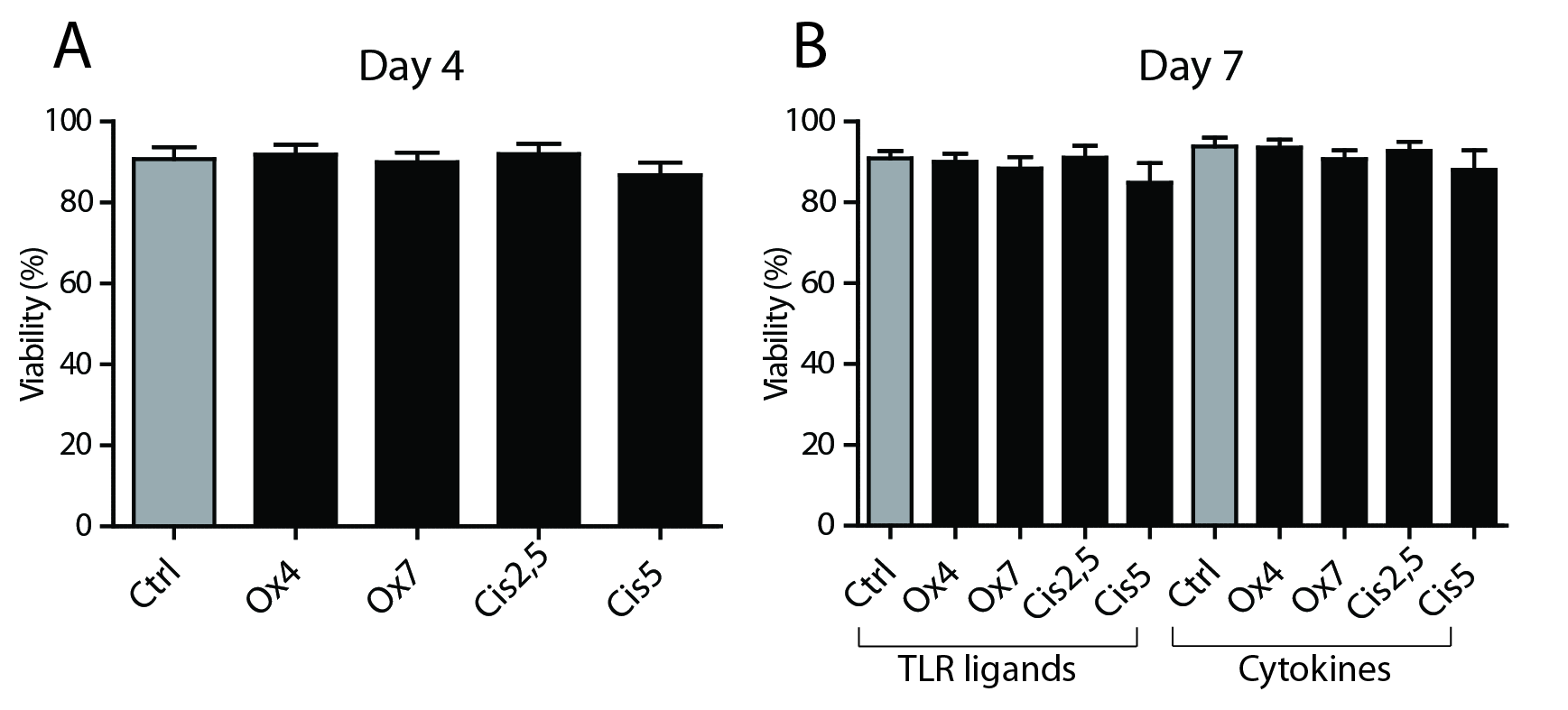
**

**Supplementary figure 3: Viability of moDCs during differentiation and maturation in the presence or absence of platinum drugs. A:** Viability of moDCs on day 4 of differentiation in the presence or absence of oxaliplatin (4 µg/ml or 7 µg/ml) or cisplatin (2,5 µg/ml or 5 µg/ml). **B**: Viability of moDCs on day 7 after maturation with TLR ligands or cytokines in the presence or absence of oxaliplatin (4 µg/ml or 7 µg/ml) or cisplatin (2,5 µg/ml or 5 µg/ml). Data are depicted as mean + SEM (n=3). MoDCs matured with TLR ligands and moDCs matured with cytokines were obtained from different donors.
